# Effects of the serine protease inhibitor rBmTI-A in an experimental mouse model of chronic allergic pulmonary inflammation

**DOI:** 10.1038/s41598-019-48577-4

**Published:** 2019-09-02

**Authors:** Ariana Corrêa Florencio, Robson S. de Almeida, Fernanda M. Arantes-Costa, Beatriz M. Saraiva-Romanholo, Adriana F. Duran, Sérgio D. Sasaki, Mílton A. Martins, Fernanda D. T. Q. S. Lopes, Iolanda F. L. C. Tibério, Edna A. Leick

**Affiliations:** 1 Department of Medicine, School of Medicine, University of São Paulo, São Paulo, SP Brazil; 2 Natural and Human Sciences Center, Federal University of ABC, Santo André, SP Brazil

**Keywords:** Chronic inflammation, Respiration, Asthma

## Abstract

To evaluate whether a recombinant serine protease inhibitor (rBmTI-A) modulates inflammation in an experimental model of chronic allergic lung inflammation. Balb/c mice were divided into four groups: SAL (saline), OVA (sensitized with ovalbumin), SAL + rBmTI-A (control treated with rBmTI-A) and OVA + rBmTI-A (sensitized with ovalbumin and treated with rBmTI-A). The animals received an intraperitoneal injection of saline or ovalbumin, according to the group. The groups received inhalation with saline or ovalbumin and were treated with rBmTI-A or saline by nasal instillation. After 29 days, we evaluated the respiratory mechanics; bronchoalveolar lavage fluid (BALF); cytokines; MMP-9, TIMP-1; eosinophils; collagen and elastic fibre expression in the airways; and the trypsin-*like*, MMP-1, and MMP-9 lung tissue proteolytic activity. Treatment with rBmTI-A reduced the trypsin-*like* proteolytic activity, the elastance and resistance maximum response, the polymorphonuclear cells, IL-5, IL-10, IL-13 and IL-17A in the BALF, the expression of IL-5, IL-13, IL-17, CD4+, MMP-9, TIMP-1, eosinophils, collagen and elastic fibres in the airways of the OVA + rBmTI-A group compared to the OVA group (p < 0.05). rBmTI-A attenuated bronchial hyperresponsiveness, inflammation and remodelling in this experimental model of chronic allergic pulmonary inflammation. This inhibitor may serve as a potential therapeutic tool for asthma treatment.

## Introduction

Asthma is a chronic inflammatory disease of the airway that is considered one of the most common chronic conditions, affecting both children and adults, and is associated with high morbidity and mortality, representing a worldwide health problem^1^.

Many cells of the innate and adaptive immune system act together with epithelial cells in asthma, causing bronchial hyperreactivity, increased mucus production, remodelling and narrowing of the airways^2^, vasodilation and plasma exudation^3^.

There is evidence that an imbalance between proteases and their inhibitors plays an important role in the development of asthma^4^. This effect can be observed more clearly in periods of exacerbation^5^.

Protein enzyme inhibitors play an important regulatory role in the cells that participate in inflammatory processes, suggesting that there is a possible use for these molecules for pharmacological purposes^6^. In respiratory disease models, some protease inhibitors have been tested, and their potential airway anti-inflammatory effects have been demonstrated^7–9^.

BmTI-A (*Boophilus microplus trypsin inhibitor*), a serine protease inhibitor extracted from the *Rhipicephalus Boophilus microplus* tick, showed inhibitory activities on bovine trypsin, human plasma kallikrein and human neutrophil elastase (HNE)^10,11^. In a mouse model of pulmonary emphysema, rBmTI-A caused a reduction in the total number of cells in the BALF, in the number of positive cells for MMP-12 and in the collagen fibre volume^12^.

The search for new effective therapies for the treatment of asthma is necessary and is a high priority, especially for asthmatic individuals whose symptoms are not well controlled by the medications that are currently available^13^. There is also a need to develop new therapeutic approaches that are capable of reversing and preventing asthma remodelling because corticosteroids do not act directly on the structural alterations of the airways^14^.

Considering the need for new therapies for asthma treatment and the great potential of serine protease inhibitors as therapeutic agents in respiratory disease models, the present study investigated the effects of rBmTI-A, a recombinant serine protease inhibitor, as a treatment for chronic allergic pulmonary inflammation in mice.

## Results

### Respiratory system mechanics

**Figure 1:**
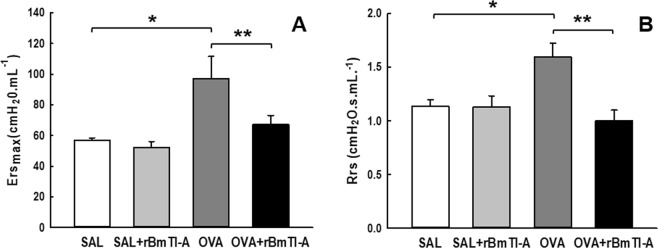
Effects of rBmTI-A treatment on the pulmonary mechanics. **(A)** Respiratory system elastance (Ers) and **(B)** respiratory system resistance (Rrs) of all experimental groups after a challenge with methacholine (300 mg/mL). Data are presented as the mean and SE. The differences were considered significant when p < 0.05. *p < 0.05 vs. control groups; **p < 0.05 vs. OVA group.

The respiratory system elastance (Ers) values for all animals are shown in Fig. 1(A). The OVA group had increased Ers values compared to those of the control groups (p < 0.05). The animals treated with the proteinase inhibitor rBmTI-A had decreased Ers values compared to those of the OVA group (p < 0.05).

Figure 1(B) presents the respiratory system resistance (Rrs) values for all the experimental groups. There was a significant increase in the Rrs of the OVA group compared to those of the control groups (p < 0.05). The group that was sensitized and received rBmTI-A (OVA + rBmTI-A) had a reduction in Rrs values compared to those of the OVA group (p < 0.05).

### Morphometric analysis

#### Eosinophils density

**Figure 2:**
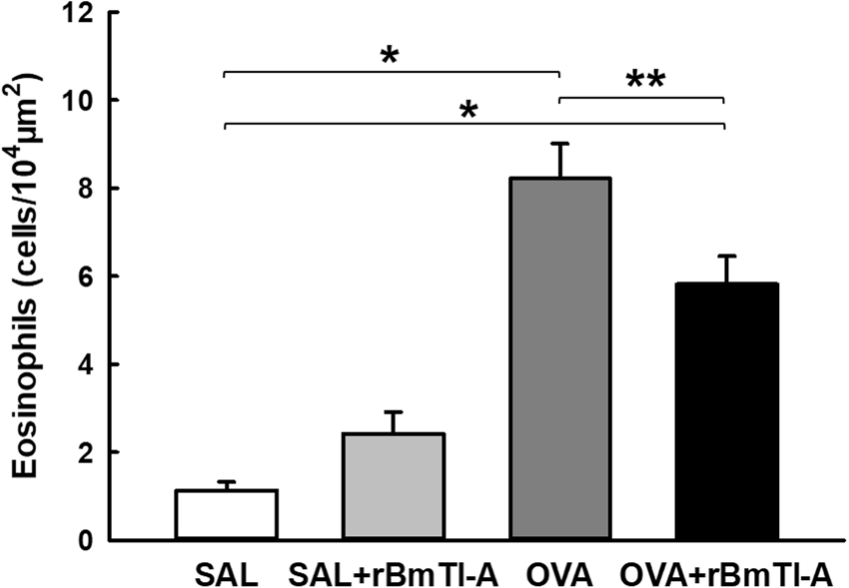
Effects of rBmTI-A treatment on eosinophil recruitment in the airway walls of all experimental groups. Data are presented as the mean and SE. The differences were considered significant when p < 0.05. *p < 0.001 vs. control groups; **p < 0.05 vs. OVA group.

The eosinophil recruitment to the airway walls is presented in Fig. 2. There was an increase in the number of eosinophils (cells/10^4^ μm^2^) in the ovalbumin-exposed animals (OVA and OVA + rBmTI-A) compared with that in the control groups (p < 0.001). The sensitized animals that were treated with rBmTI-A (OVA + rBmTI-A) had a reduction in the number of eosinophils compared to that of the OVA group (p < 0.05).

#### Extracellular matrix remodelling

**Figure 3:**
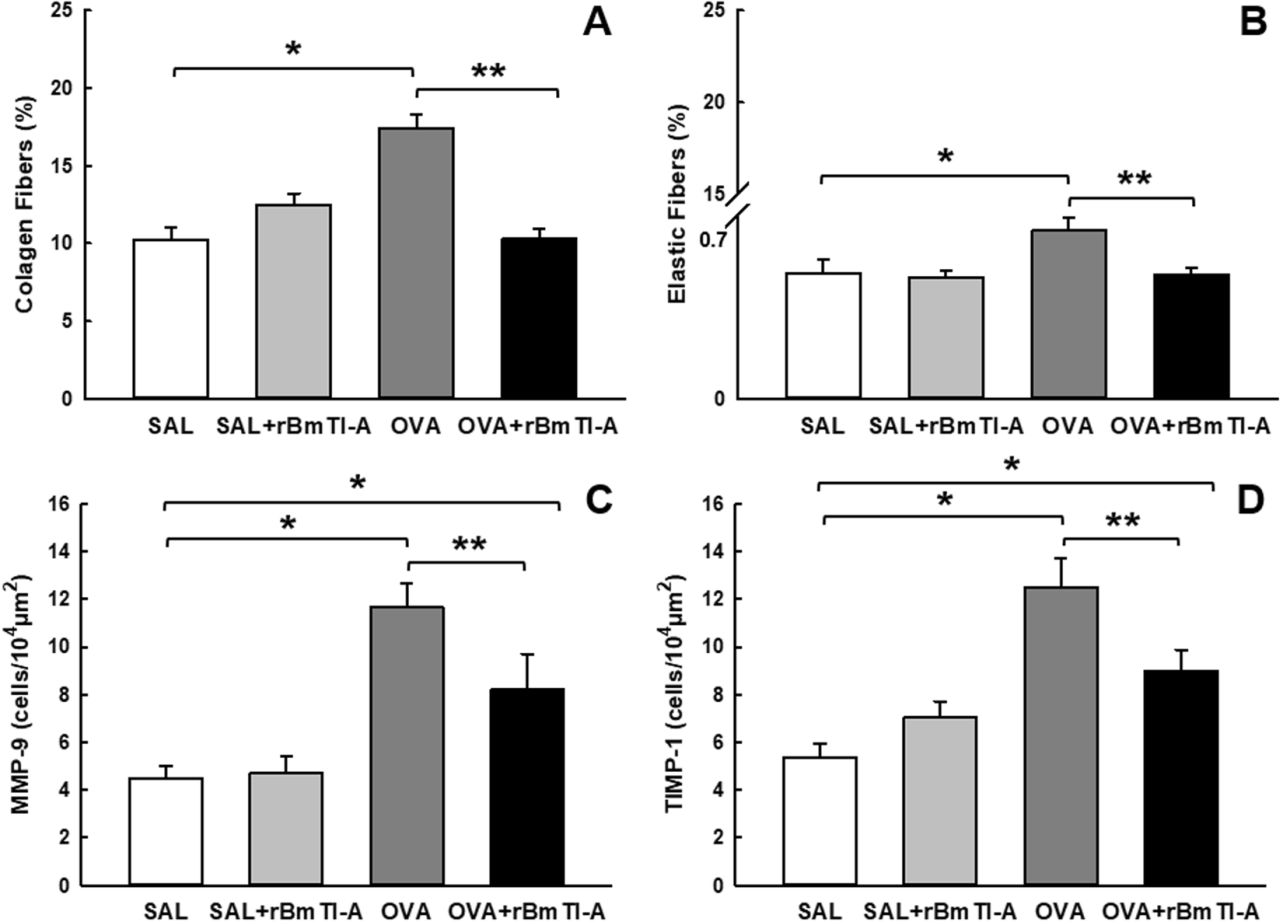
Effects of rBmTI-A treatment on extracellular matrix remodelling. **(A)** Collagen fibres and **(B)** elastic fibre volume fractions in the airway walls. **(C)** MMP-9 and **(D)** TIMP-1 expression in the airway walls of the four experimental groups. Data are presented as the mean and SE. The differences were considered significant when p < 0.05. *p < 0.05 vs. control groups; **p < 0.05 vs. OVA group.

Figure 3(A,B) show the volume fractions of collagen and elastic fibres in the airway walls, respectively. There was an increase in the volume fraction of the collagen and elastic fibres (p < 0.05) in the airway walls of the OVA group compared to those in the control groups. Compared with the OVA group, treatment with the inhibitor rBmTI-A reduced the fractions of collagen and elastic fibres (p < 0.05) in the airway walls.

The MMP-9- and TIMP-1-positive cells in the airway walls are shown in Fig. 3(C,D). The numbers of MMP-9- and TIMP-1-positive cells were greater in the OVA group compared with those in the control groups (p < 0.05). The OVA + rBmTI-A group had a decreased number of MMP-9- and TIMP-1-positive cells compared to those in the OVA group (p < 0.05).

#### Lung inflammation

**Figure 4:**
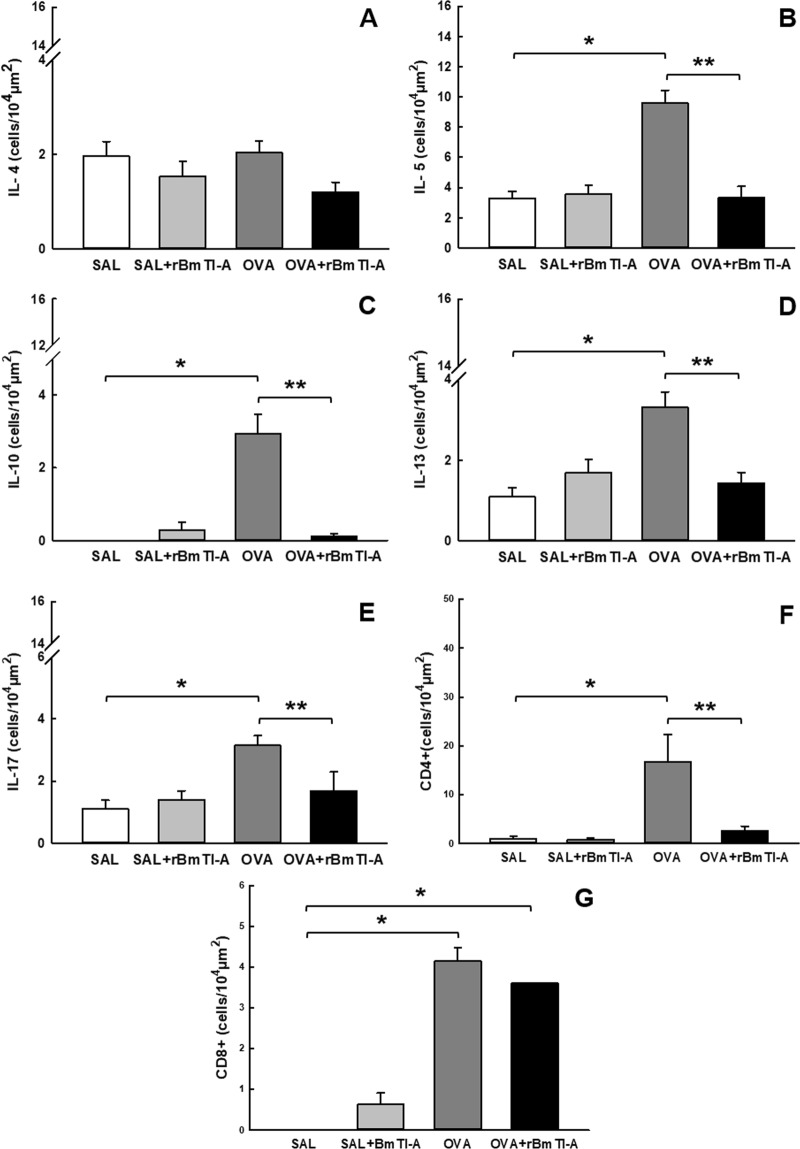
Effects of rBmTI-A treatment on the inflammatory response. **(A)** IL-4, **(B)** IL-5, **(C)** IL-10, **(D)** IL-13, **(E)** IL-17, **(F)** CD4+ and **(G)** CD8+ positive cells in the airway walls in all experimental groups. Data are presented as the mean and SE. The differences were considered significant when p < 0.05. *p < 0.05 vs. control groups; **p < 0.05 vs. OVA group.

The inflammatory cells in the airway walls of the four experimental groups are shown in Fig. 4(A) (IL-4), 4(B) (IL-5), 4(C) (IL-10), 4(D) (IL-13), 4(E) (IL-17), 4(F) (CD4^+^) and 4(G) (CD8^+^). The IL-5, IL-10, IL-13, IL-17, CD4+ and CD8 + positive cells were increased in the OVA group compared to those in the control groups (p < 0.05). The sensitized animals that were treated with the serine protease inhibitor (OVA + rBmTI-A group) showed decreased IL-5, IL-10, IL-13, IL-17 and CD4+ positive cells in the airway walls compared to those in the OVA group (p < 0.05).

#### Bronchoalveolar lavage fluid (BALF)

**Figure 5:**
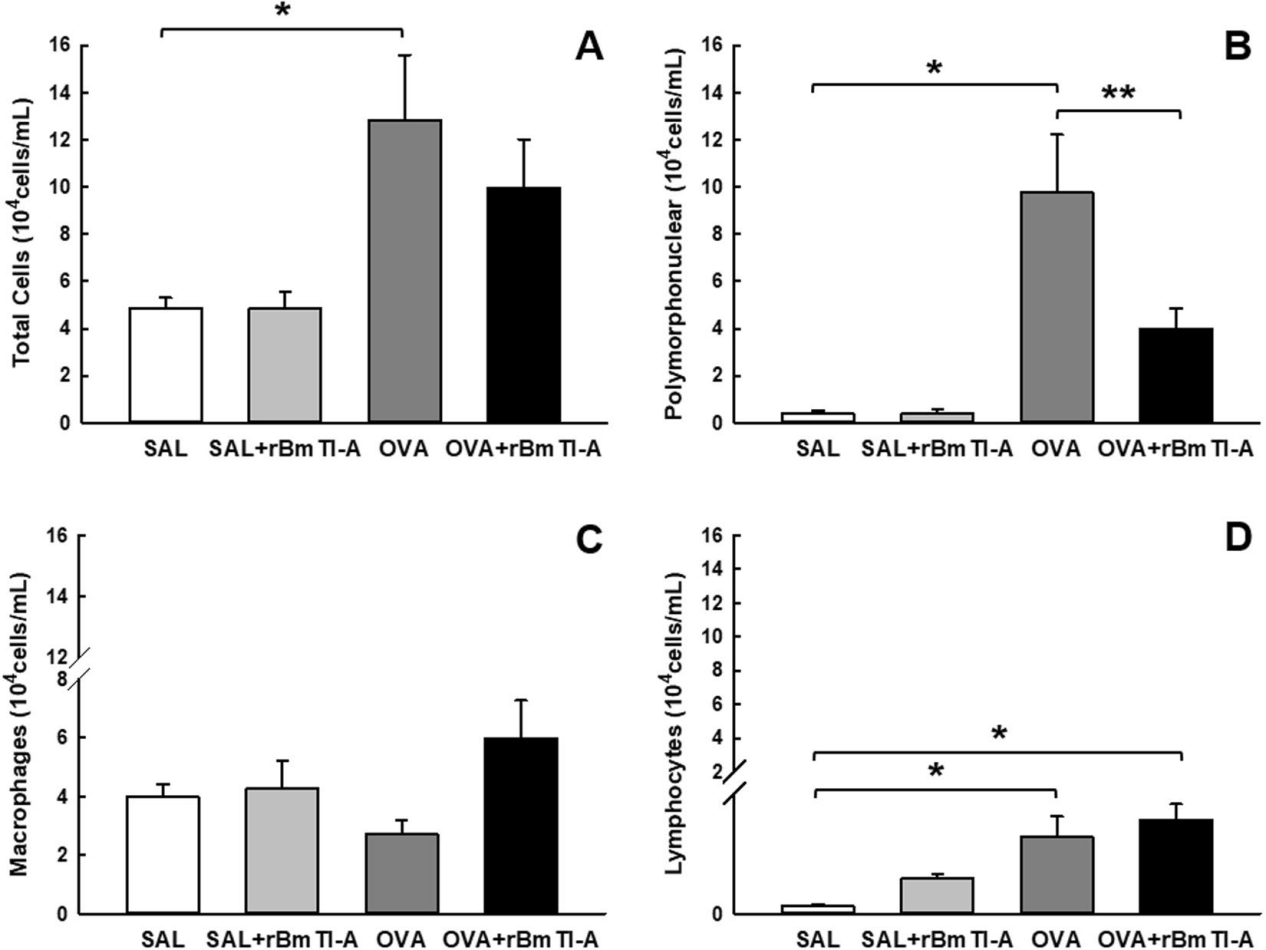
Effects of rBmTI-A treatment on the BALF inflammatory cells. **(A)** Total cells, **(B)** polymorphonuclear cells, **(C)** macrophages and **(D)** lymphocytes in all experimental groups. Data are presented as the mean and SE. The differences were considered significant when p < 0.05. *p < 0.05 vs. control groups; **p < 0.05 vs. OVA group.

The inflammatory cells in the BALF samples from all experimental groups are shown in Fig. 5(A) (total cells), 5(B) (polymorphonuclear cells), 5(C) (macrophages) and 5(D) (lymphocytes). In the OVA group, we found an increase in the number of total cells and of polymorphonuclear cells (p < 0.05) compared to those in the control groups. The number of polymorphonuclear cells from the sensitized group was reduced by the treatment in the OVA + rBmTI-A group compared to that in the OVA group (p < 0.05).

**Figure 6:**
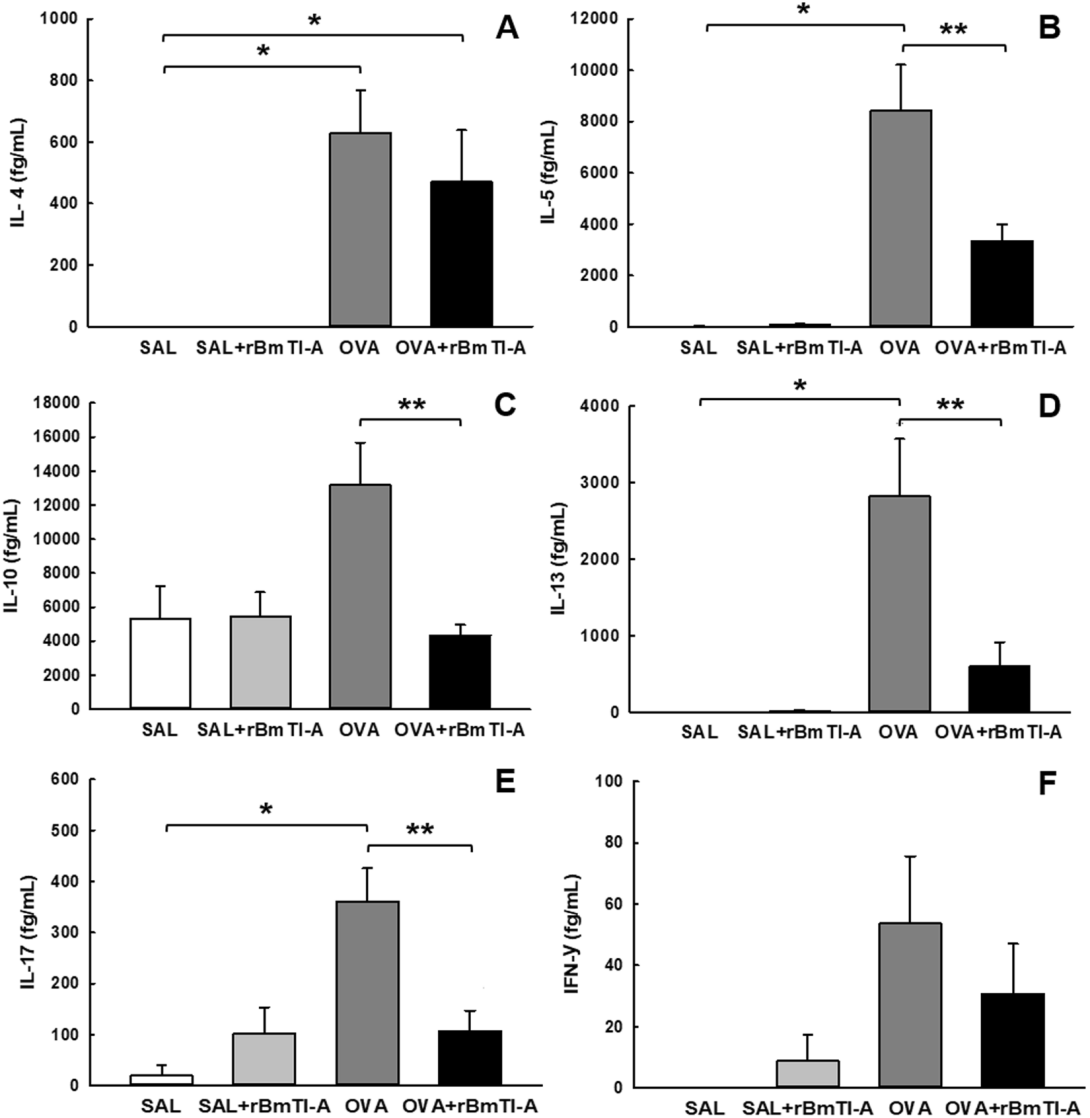
Effects of rBmTI-A treatment on the BALF inflammatory markers. **(A)** IL-4, **(B)** IL-5, **(C)** IL-10, **(D)** IL-13, **(E)** IL-17A and **(F)** INF-γ levels in all experimental groups. Data are presented as the mean and SE. The differences were considered significant when p < 0.05. *p < 0.05 vs. control groups; **p < 0.05 vs. OVA group.

The cellular inflammation markers are presented in Fig. 6(A) (IL-4), 6(B) (IL-5), 6(C) (IL-10), 6(D) (IL-13), 6(E) (IL-17A) and 6(F) (INF- γ**)** The concentrations of IL-4, IL-5, IL-13 and IL-17A in the OVA group were higher than those in the control groups (p < 0.05). Treatment with the serine protease inhibitor reduced the concentrations of IL-5, IL-10, IL-13 and IL-17A in the OVA + rBmTI-A group compared to those in the OVA group (p < 0.05).

### Proteolytic Activity in pulmonary homogenate

**Figure 7:**
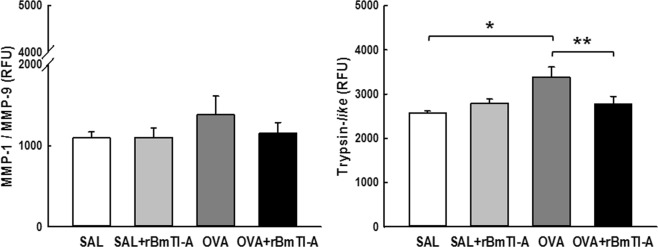
Effects of rBmTI-A treatment on the MMP1-1/MMP-9 **(A)** and trypsin-*like* **(B)** proteolytic activity in the pulmonary homogenate of all experimental groups. Data are presented as the mean and SE. The differences were considered significant when p < 0.05. *p < 0.05 vs. control groups; **p < 0.05 vs. OVA group.

There were not significant differences among the groups in the proteolytic activity of MMP-1 and MMP-9, as depicted in Fig. 7(A).

Figure 7(B) shows the trypsin-*like* serine protease proteolytic activity for all the experimental groups. There was an increase in the trypsin-*like* activity in the OVA group compared to that in the control groups (p < 0.05). The sensitized group that was treated with the inhibitor rBmTI-A showed a reduction in this activity compared to that in the OVA group (p < 0.05).

### Qualitative analysis

**Figure 8:**
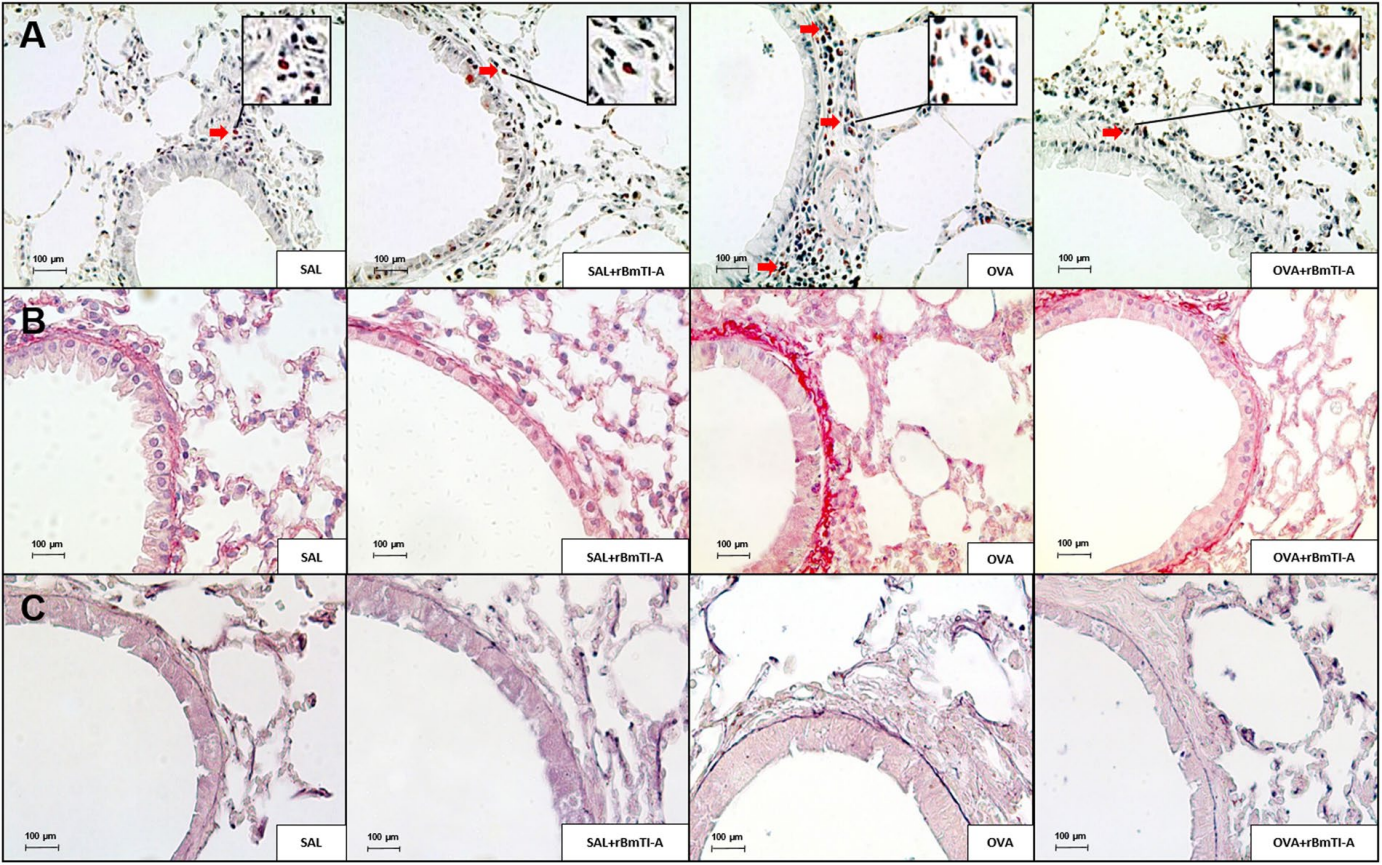
Representative photomicrographs of mouse lung airway walls from all experimental groups. The sections underwent specific staining for the detection of **(A)** eosinophils (stained red); **(B)** collagen fibres (stained red); and **(C)** elastic fibres (stained deep purple). 400× magnification. The red arrows indicate positive cells. The inserts with larger magnification show the positive cells in detail.

**Figure 9:**
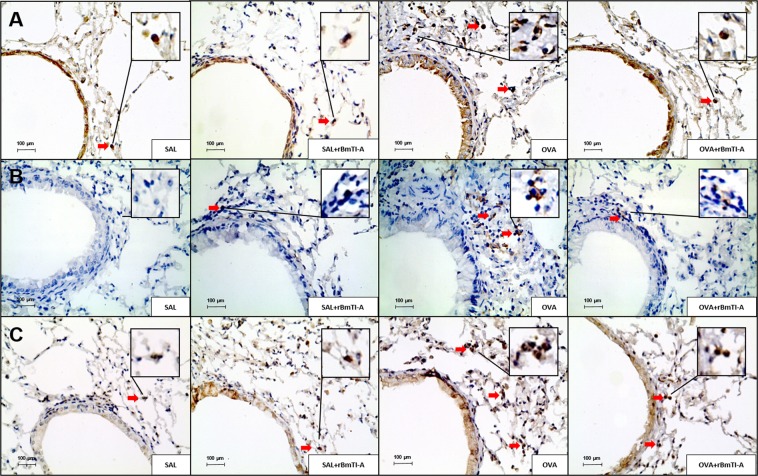
Inflammatory and extracellular matrix remodelling markers in the airways of all experimental groups. **(A)** IL-5; **(B)** IL-10; **(C)** TIMP-1. 400× magnification. Positive cells are stained brown, and the red arrows indicate positive cells. The inserts with larger magnification show the positive cells in detail.

Representative airway photomicrographs from the four experimental groups are shown in Figs 8 and 9. The sections were stained with Luna for the detection of eosinophils (Fig. 8(A)); with picrosirius for the detection of collagen content (Fig. 8(B)); and Weigert’s resorcin-fuchsin for the detection of the elastic fibre content (Fig. 8(C)). Figure 9 shows the quantification by immunohistochemistry of the cytokine expression (A–C) and of the metalloproteinase inhibitor expression (TIMP-1, (D)).

We observed an increase in the number of eosinophils, collagen and elastic fibre content and in the IL-5, IL-10 and TIMP-1 airway expression in the OVA group compared to those in the control groups (SAL and SAL + rBmTI-A). Treatment with rBmTI-A attenuated these increases in the OVA + rBmTI-A group compared to that in the OVA group.

## Discussion

In the present study, we evaluated the effect of the serine protease inhibitor rBmTI-A in an experimental model of chronic allergic pulmonary inflammation. We demonstrated that treatment with rBmTI-A reduced the trypsin-*like* proteolytic activity in the pulmonary homogenate. Compared to those of the controls, this result, in the animals that were sensitized with ovalbumin and that were treated with rBmTI-A, was associated with the attenuation of airway hyperresponsiveness after challenge with the bronchoconstrictor methacholine; the reduction of eosinophil numbers in the BALF and in the airways; decreased expression of Th2/Th17 (IL-5, IL-10, IL-13 and IL-17) inflammatory cytokines in the BALF and in the airways; the reduction of CD4+ positive cells in the airways; attenuation of the MMP-9 and TIMP-1 expression in the airways; and the decrease of the extracellular matrix remodelling, as evaluated by the reduction of the collagen and elastic fibre content in the airways.

Experimental models of murine chronic allergic lung inflammation have been widely used because they reproduce important characteristics of the immune and inflammatory response in asthma^15,16^. The Balb/c mice used in our experimental model also have good Th2 profile immunological response^17^ and good reactivity to methacholine^18^.

A previous study demonstrated that the administration of rBmTI-A had as much of a protective effect against the development and progression of pulmonary emphysema as the effect of treatment after the development of the disease, with a decrease in the distal air spaces in treated animals. This was the first study using animals that were treated with rBmTI-A. This study compared the effect of the treatment with one dose of rBmTI-A (35.54 pmol in 50 μL of saline solution) to the effect of treatment with two doses of rBmTI-A (same concentration), with one dose at the beginning of the sensitization protocol and another at the end^12^. Based on this study, we designed our treatment protocol.

There is evidence that the imbalance between proteases and their inhibitors plays an important role in the development of asthma^4^, and that this effect is more pronounced in the periods of disease exacerbation^5^.

The participation of trypsin-*like* serine proteases in asthma, such as human plasma kallikrein (HuPK)^19^, neutrophil elastase^4^ and trypsin^20^, has been reported in some studies. The role of neutrophil elastase and trypsin in asthma is even more evident in the presence of a deficiency of alpha-1 antitrypsin, a known serine protease inhibitor, which is able to inhibit some enzymes, such as trypsin, neutrophil elastase and protease-3^21^.

Several trypsin-*like* serine protease inhibitors have been studied as therapeutic tools for the treatment of several pathologies, such as thrombosis, asthma and COPD^22^.

Some studies have shown the BmTI-A inhibitory activity for HuPK, neutrophil elastase and trypsin^10,11,23^. These findings corroborate the results that we obtained using recombinant BmTI-A since we observed a reduction of the trypsin-*like* proteolytic activity in the pulmonary homogenate of animals that were sensitized and treated with rBmTI-A compared to that in the sensitized group that did not receive the inhibitor.

Experimental models of chronic allergic inflammation showed a significant elevation of the Ers and Rrs when treated with an intravenous administration of methacholine^18,24^. In the present study, the animals that were sensitized and treated with the serine protease inhibitor rBmTI-A showed a reduction of approximately 30% in the Ers and 37% in the Rrs when compared to those in the sensitized and untreated groups. Other serine protease inhibitors have also showed the ability to attenuate these pulmonary responses; for example, the *CrataBL* inhibitor reduced the Ers and Rrs values in mice with chronic allergic pulmonary inflammation^25^. Three inhibitors of serine proteases, FOY, FUT and UTI, reduced the Ers and Rrs values in a model of chronic allergic pulmonary inflammation^26^. Saw and Arora (2015) also observed a reduction of the pulmonary resistance in murine with airway allergic inflammation that were treated with the serine protease inhibitor AEBSF^27^.

Remodelling is related to the decreased lung function in individuals with asthma and in asthmatic animal models and may be a determining factor for hyperresponsiveness^16^. In asthma, the remodelling process involves the deposition of collagen and elastic fibres^28^; MMP-9 is one of the main metalloproteases involved in this process, as it is capable of degrading many types of collagen, gelatin, elastin proteins, fibronectin and other extracellular matrix components^14^, and MMP-9 is directly related to the severity of asthma^29^.

The inhibitor rBmTI-A effectively attenuated remodelling, decreasing the percentage of collagen and elastic fibres in the airways of the sensitized animals compared to those in the controls. In a murine model of elastase-induced pulmonary emphysema, rBmTI-A also reduced the percentage of collagen and elastic fibres in the pulmonary parenchyma^30^; however, in a previous study with a different experimental model, rBmTI-A attenuated only the percentage of collagen fibres^12^. The *CrataBL* serine protease inhibitor also reduced the fraction of collagen and elastic fibres in a murine asthma model^12^. Another protease inhibitor, EcTI, also attenuated the deposition of these fibres in a pulmonary emphysema model^9^.

In association with the increase of collagen and elastic fibres in the airways of the OVA group, we observed an increase in the expression of MMP-9. The sensitized animals that received rBmTI-A showed a decrease in airway MMP-9 expression.

The regulation of MMP-9 secretion in the airways is complex since several inflammatory and structural cells are able to produce this protease^31^. The activation of MMPs involves other MMPs and serine proteases, which may explain the reduction of MMP-9 release in the sensitized group that received the rBmTI-A serine protease inhibitor^32^. The protease-activated receptor (PAR-2) releases MMP-9 through the airway epithelial cells and is activated primarily by serine proteases such as trypsin and tryptase^33^. Considering that there was a decrease in the trypsin-*like* proteolytic activity in the pulmonary homogenate of the sensitized animals that received rBmTI-A compared to that of the controls, possibly also occurred a reduction of PAR-2 activation by trypsin and a consequent decrease in the release of MMP-9, deposition of collagen and elastic fibres observed in this same group.

One of the regulatory mechanisms of MMPs is the balance between the proteolytic/anti-proteolytic activity since the deregulation of MMPs and their endogenous tissue inhibitors, TIMPs, plays an important role in tissue remodelling processes^34^. Righetti *et al*.^35^ showed an increase in the MMP-9-specific metalloprotease inhibitor TIMP-1 in asthma, as was also observed in our experimental model. In addition, treatment with rBmTI-A attenuated the increase in TIMP-1^35^. This attenuation probably occurred in association with the reduction of MMP-9 expression in the sensitized and rBmTI-A treated groups, maintaining the balance of the proteolytic activity in this group. This decrease was also observed in another study using the same experimental asthma model that was treated with the *CrataBL* serine protease inhibitor^25^.

Our data also showed that there was an increase in the eosinophilic infiltrate in the airways of animals that were sensitized with ovalbumin. Notwithstanding, treatment with rBmTI-A significantly attenuated this response in the animals sensitized and treated with the inhibitor. The *CrataBL* serine protease inhibitor also reduced the eosinophilic infiltrate in the airways of mice that were sensitized with ovalbumin^25^. This effect was also observed in response to treatment with the inhibitors FOY, FUT and UTI in another model of chronic allergic pulmonary inflammation^26^.

Eosinophilic inflammation is increased in several asthma models^16,36,37^. In humans, there is evidence that even in mild forms of asthma, there is persistent chronic inflammation of the airways, with increasing numbers of eosinophils and other inflammatory cells^3^.

In the bronchoalveolar lavage fluid(BALF) cell evaluation, as observed in other studies, the numbers of total cells, polymorphonuclear cells and lymphocytes were increased in the BALF of the sensitized group compared to those in the control groups^16,25,38^.

The increased number of polymorphonuclear cells in the BALF from the OVA group was probably due to the increase in eosinophils since in the analysis of the tissues with eosinophil-specific staining (LUNA), we observed that the cells around the airways were mostly eosinophils. An increase in the number of eosinophils in the BALF has been consistently reported among asthmatic individuals, and the increase correlates with the severity of the disease^39^.

In the group that was sensitized and treated with rBmTI-A, there was a reduction in the number of polymorphonuclear cells in the BALF. However, rBmTI-A treatment did not attenuate the lymphocyte response, as in another study that evaluated the effect of rBmTI-A on pulmonary emphysema in a murine model^12^. The reduction in the eosinophil content in the sensitized murine BALF was also observed in studies that used other serine protease inhibitors, such as *CrataBL*^25^, Nafamostat Mesylate^40^, FOY, FUT, UTI^26^ and the AEBSF inhibitor^27^.

We evaluated the concentration of cytokines in the BALF and in the airways, and in both analyses, we observed a reduction in IL-5, IL-10, IL-13 and IL-17 in the animals that were sensitized and treated with rBmTI-A compared to those in the OVA group. Studies that were performed with other serine protease inhibitors have shown reduced levels of these cytokines in a murine model of airway inflammation that was treated with other serine protease inhibitors. The serine protease inhibitors FOY, FUT and UTI reduced the concentration of IL-5, IL-6, IL-13 and IL-17 in BALF in an experimental model of chronic allergic inflammation in mice^26^. Others studies have reported the reduction of the IL-4, IL-5 and IL-13 concentration in the murine BALF of sensitized mice that were treated with *CrataBL* and AEBSF inhibitors^25,27^, and Ishizaki *et al*.^40^ showed that there was a reduction in IL-13 in a group of animals that was sensitized and treated with Nafamostat Mesylate^40^.

IL-5 is a cytokine that is essential for the growth, maturation, activation, and suppression of eosinophil apoptosis^41^. IL-5 has also been implicated in the induction of hyperresponsiveness^42^. Data have demonstrated that the inhibition of IL-5 effectively reduces exacerbation, which occurs often in severe asthma, especially in the eosinophilic pathogenesis subgroup^43^.

The identification of Th2/Th17 cells in allergic asthma led to the observation that different clinical phenotypes can coexist in the same patient and are related to greater severity of the disease^38^. Th2 cytokines (IL-4 and IL-13) induce remodelling and hyperresponsiveness in asthma^44^.

In some murine airway inflammation models, IL-17 controls airway hyperresponsiveness and remodelling^45^ and induces the resistance of bronchial epithelial cells to the effects of steroids^46^.

IL-10 is different from the cytokines mentioned above and is known for its anti-inflammatory role, as IL-10 is capable of inhibiting various cytokines^47^. Nonetheless, IL-10 is also paradoxically associated with hyperresponsiveness and IL-5 production^48^.

The effect of rBmTI-A on cytokine levels can be attributed to the role of trypsin-*like* serine proteases in the activation of the PAR-1 and PAR-2 protease-activated receptors, which appear to be involved in the production and release of these inflammatory components. According to Shigetomi *et al*.^49^, the effects of basic pancreatic trypsin (BPTI) are mediated by anti-inflammatory mechanisms through the protection of the high affinity thrombin receptor and the protease-activated receptor PAR-1. PAR-1 can be activated by trypsin, thrombin and matrix-1 metalloproteinase (MMP-1), which leads to the production of various proinflammatory cytokines and chemokines. Other studies have observed a reduction in hyperresponsiveness, eosinophilia and IL-2, IL-3, IL-4, IL-5, IL-6, IL-7, IL-13 and IL-7 in asthma models that received PAR-2 inhibitors and PAR-2 signalling blockers^50^.

The increase in CD4+ positive cells in the airways of the sensitized group was attenuated by treatment with the serine protease inhibitor rBmTI-A. Activated T cells, especially CD4+ T cells, are protagonists in the pathophysiology of allergic asthma, modulating the production of cytokines^51^.

Cytokines play an essential role in the development and regulation of asthma, which explains the importance of the effect of rBmTI-A on the inflammatory process.

This study has some limitations. For example, rBmTI-A was tested in an experimental model of chronic allergic pulmonary inflammation. Therefore, we cannot extrapolate our findings directly to humans. In addition, we did not evaluate the toxicity of the inhibitor.

The results obtained in this study showed that the rBmTI-A serine protease inhibitor was effective in attenuating the changes in lung mechanics, inflammation, and remodelling of the airways in this animal model of chronic allergic pulmonary inflammation. Although more studies need to be performed to elucidate the mechanisms responsible for these changes, this inhibitor appears to be a possible pharmacological tool for the treatment of asthma.

## Conclusion

In this experimental model of chronic allergic pulmonary inflammation, we can conclude that the rBmTI-A serine protease inhibitor was effective in reducing the following responses:

- Proteolytic activity of trypsin-*like* proteases in the pulmonary homogenate;
- Maximum response of elastance and resistance of the respiratory system;
- The number of eosinophils in the airways;
- The number of polymorphonuclear cells in the bronchoalveolar lavage fluid;
- The concentration of the IL-5, IL-10, IL-13, IL-17A cytokines in the bronchoalveolar lavage fluid;
- The expression of IL-5, IL-10, IL-13, IL-17, CD4+, MMP-9 and TIMP-1 in the airways; and
- The fraction of collagen and elastic fibres of the airways.

Therefore, our results showed that the rBmTI-A serine protease inhibitor was effective in reducing methacholine hyperresponsiveness, airway inflammation and remodelling in this experimental model of chronic allergic pulmonary inflammation. Although more studies need to be performed, this inhibitor may contribute as a potential therapeutic tool for the treatment of asthma.

## Methods

The animals (male *Balb/c* mice) used in this study initially weighed 25–30 g and were approximately 6–8 weeks old. All the mice received humane care in compliance with the “Guide for the Care and Use of Laboratory Animals” (NIH publication 85–23, revised 1985), and all the experiments described in this study were approved by the institutional review board of the University of Sao Paulo (Sao Paulo, Brazil).

### Experimental groups and study design

**Figure 10:**
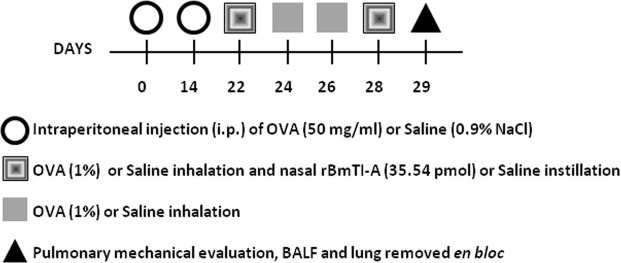
Timeline of the experimental protocol. On the first and fourteenth day of the protocol, the animals received intraperitoneal injections with ovalbumin or saline solution. On days 22, 24, 26 and 28, the animals received ovalbumin or saline by aerosol inhalation. On days 22 and 28, 2 hours after inhalation, the animals received an intranasal instillation with saline (SAL and OVA) or with rBmTI-A (SAL + rBmTI-A and OVA + rBmTI-A). On the twenty-ninth day, the animals were anaesthetized, tracheostomized and the evaluation of the pulmonary mechanics and BALF were performed; the lungs were removed to perform the other analyses.

The animals were randomly divided into the following 4 groups of 8 animals each, according to the protocol (Fig. 10):

(A) SAL: animals that received saline solution (0.9% NaCl) by inhalation and nasal instillation;
(B) SAL + rBmTI-A: animals that received saline solution (0.9% NaCl) by inhalation with and rBmTI-A by nasal instillation;
(C) OVA: animals that received ovalbumin solution by inhalation; and
(D) OVA + rBmTI-A: animals that received ovalbumin solution by inhalation and rBmTI-Aby nasal instillation.

### Experimental model of chronic allergic pulmonary inflammation

Mice received intraperitoneal injection (i.p.) with a solution of 50 μg ovalbumin grade III (Sigma - Aldrich) and 6 mg of aluminium hydroxide (Pepsamar ®, Sanofi - Synthelabo SA, Rio de Janeiro, Brazil) in a total volume of 0.2 ml on days 0 and 14. On days 22, 24, 26 and 28, the animals were placed in an acrylic display box (30 × 15 × 20 cm) coupled to an ultrasonic nebulizer (Pulmoclear II, Soniclear, Sao Paulo, Brazil) and underwent an aerosol inhalation of OVA diluted in NaCl 0.9% (saline) at a concentration of 10 mg/mL (1%) for 30 minutes. The control groups received saline solution (NaCl 0.9%) and aluminium hydroxide (6 mg) i.p., and on the days of the inhalation challenges control animals were exposed to aerosol saline 0.9%, for 30 minutes.

### Inhibitor expression and purification

The cloning of the recombinant inhibitor, rBmTI-A, used in this design was performed with an *Rhipicephalus Boophilus microplus tick* intestine cDNA library by the constructing of the recombinant vector rBmTI-A/pPIC9K, as described by Sasaki and Tanaka (2008)^52^.

The rBmTI-A purification was carried out using affinity chromatography with a trypsin-Sepharose column that had been previously equilibrated with 0.05 M Tris-HCl buffer, pH 8.0. Subsequently, 0.05 M Tris-HCl buffer containing 0.2 M NaCl, pH 8.0, was passed through the column until the collected material reached an absorbance reading of 0.05 at 280 nm with a spectrophotometer. The material was then eluted using a 0.5 M KCl/HCl solution, pH 2.0. The eluted fractions were immediately neutralized with 1 M Tris–HCl buffer, pH 8.0. The samples with high absorbance were pooled, and their concentration and inhibitory constants (Ki) were determined. rBmTI-A was further purified by gel filtration chromatography with a Sephadex 75 column (ÄKTA purifier System, GE Healthcare), and equilibration and elution were performed with PBS buffer^52^.

### Treatment protocol

Mice were treated by intranasal instillation 2 hours after the inhalation at the first and last inhalation (days 22 and 28). Animals received a nasal instillation of 35.54 pmol of the recombinant inhibitor (rBmTI-A) in 50 μl of saline solution (0.9% NaCl)^12^, following the protocol shown in Fig. 10. The control animals received 50 μl of saline solution (0.9% NaCl) by nasal instillation.

### Respiratory mechanics evaluation

Thirty days after the initiation of the sensitization protocol (twenty-four hours after the last inhalation challenge), the animals were anaesthetized with thiopental (80 mg/kg, i.p.) and were tracheostomized. The mice were mechanically ventilated in an acrylic plethysmograph (120 cycles/min, 10 mL/kg) connected to a Harvard 683 small animal ventilator (Harvard Apparatus, Massachusetts, USA). Tracheal pressure signals and lung volume were measured with differential pressure transducers (Honeywell 163PC01D36, Freepot, IL) and converted by an analogue digital board (DT01EZ, Data Translation, Marlboro, MA). The values of elastance (Ers) and resistance (Rrs) of the respiratory system were calculated using the equation of motion of the respiratory system, Ptr (t) = Rrs. V′(t) + Ers. V (t) where t represents time, Ptr represents tracheal pressure, V′ represents airflow and V represents lung volume. Basal and maximal Rrs and Ers values were calculated after methacholine aerosol administration (3, 30 and 300 mg/mL for 1 minute - Dose-Response Curve).

### Bronchoalveolar lavage fluid

Bronchoalveolar lavage fluid (BALF) collection was performed by washing the lungs with three 0.5 mL PBS injections through the tracheal cannula. The recovered volume was approximately 90% of the volume injected. This procedure was repeated three times.

#### Total and differential balf cell counts

The collected BALF was centrifuged at 800 revolutions per minute (rpm) for ten minutes at 4 °C. The cell pellet was resuspended in 0.3 ml of 0.9% sterile saline solution. From this solution, 20 μl was removed for total cell analysis using a *Neubauer* haemocytometer chamber and optical microscope at 1000x magnification. The differential cell counts were performed using 100 μl of the supernatant, which was centrifuged (450 rpm for 10 minutes) to generate the slides. The slides were stained with the Diff-Quick reagent. The differential cell count was determined with 300 cells/slide with an optical microscope.

#### Determination of the cytokine concentration by flow cytometry (cytometric bead array - CBA)

The cytokine levels in the BALF was measured using the CBA method with CBA Mouse Enhanced Sensitivity Flex Set (BD Pharmingen) kits specific for the cytokines IL-4, IL-5, IL-10, IL-13, IL-17A and INF-γ according to the manufacturer’s instructions. Samples were incubated with capture microspheres with different fluorescence intensities that were coated with a capture antibody specific for each cytokine. Thereafter, a second incubation was performed with high sensitivity detection antibodies labelled with phycoerythrin (PE), which emits a fluorescent signal that is proportional to the amount of protein. After the incubations, 1 mL of the wash solution was added, and the material was centrifuged for 10 minutes at 1100 rpm. The supernatant was discarded, and in 300 μL of the wash buffer, the samples were resuspended for the analysis of the complexes with a flow cytometer (LSRFortessa - BD Biosciences). The results were analysed with the program BD FCAPArray 3.0 (BD Biosciences) and are displayed in a graphic format.

### Morphometric analysis

The left lung was fixed with 10% formalin and, after twenty-four hours, was transferred to 70% ethanol. After fixation, the lung was cut on its largest axis, was paraffin embedded and 4 μm thick histological sections were cut and stained with LUNA (for eosinophil analysis), picrosirius (for the analysis of the collagen fibres), Weigert’s resorcin-fuchsin (for the analysis of elastic fibres); the sections were also used for immunohistochemical evaluation. Morphometric analysis was performed using an optical microscope (CH30, Olympus, Japan), and approximately three fields of three to five airways of each animal were randomly selected and were evaluated^36,53–55^.

#### Measurement of eosinophil density

To evaluate the eosinophil density, the LUNA technique was used, which identifies eosinophil granules. The point counting technique^56^ was used with a reticulum of a 100 points/50 intercepts grid, which was attached to the microscope eyepiece and superimposed on the tissue (10^4^ mm^2^ total area). The number of positive cells in the airway wall was calculated as the number of positive cells in each field divided by the number of points coinciding with the area of tissue present in the same field of the reticulum (10^4^ mm^2^).

#### Volume Fraction of the collagen and elastic fibres

Picrosirius staining was used to quantify collagen fibres, and resorcin-fuchsin staining was used to identify the elastic fibres in the airways. The measurement of the optical density was used for the morphometric analysis of the collagen and elastic fibres in the airway wall. The images were obtained using a Leica DM4000B microscope (*Leica Microsystems, Wetzlar, Germany*) and a digital camera Leica DFC420 (*Leica Microsystems, Wetzlar, Germany*) that were connected to a computer using the Leica Qwin software (*Leica Microsystems, Cambridge, England*); the images were acquired at 400× magnification. The images were processed by the ImageProPlus software (*Media Cybernetics, Bethesda, MD*), which allows the user to set a threshold of colour tones that represents the positive areas, in order to quantify a predetermined area. Two binary-colour thresholds were defined: one for the fibre calculation (according to staining) and one for the airways. Thus, the proportion of the collagen or elastic fibres per airway area was quantified. The results are expressed as the percentage of the positive area (volume fraction).

#### Immunohistochemical evaluation

For the immunohistochemical evaluation, the following antibodies were used: anti-IL-4 (Santa Cruz Biotechnology, California, USA; 1:600), anti-IL-5 (Santa Cruz Biotechnology, California, USA; 1:100), anti-IL-10 (Santa Cruz Biotechnology, California, USA; 1:500), anti-IL-13 (Santa Cruz Biotechnology, California, USA; 1:700), anti-IL-17 (Santa Cruz Biotechnology, California, USA; 1:800), anti-CD4+ (Santa Cruz Biotechnology, California, USA; 1:25), anti-CD8+ (Santa Cruz Biotechnology, California, USA; 1:50), anti-MMP-9 (Santa Cruz Biotechnology, California, USA; 1:500) and anti-TIMP-1 (Santa Cruz Biotechnology, California, USA; 1:100).Immunohistochemistry was performed with the following sequence of procedures: antigenic recovery, endogenous peroxidase blockade and nonspecific binding blockade, incubation with the primary antibody, incubation with the secondary antibody and complex, counterstaining and assembly of the blades. The count was determined as described above for the evaluation of eosinophil density.

### Evaluation of the pulmonary homogenate proteolytic activity

The right lung was homogenized in PBS (600 μL) using metal beads, as recommended by the manufacturer (*Powerlyzer, MoBio Laboratories Inc., USA*).

Pulmonary homogenate was used in an enzymatic assay to analyse the proteolytic activity of MMP-1, MMP-9 and trypsin-like in lung tissue according to the following protocol. For the analysis of MMP-1 and MMP-9 activity, an incubation of 10 μL of the sample (pulmonary homogenate) with 2 μL of the fluorogenic substrates for MMP-1/MMP-9 (MMP-1/MMP-9 substrate – *Calbiochem*, San Diego, CA) and 88 μL of buffer (0.1 M Tris-HCl, 0.15 M NaCl and 0.1 M Triton X-100) at pH 8.0 was performed. For the analysis of trypsin-like proteolytic activity, 10 μL of sample (pulmonary homogenate), 4 μL of the fluorogenic trypsin-like substrate (*Z-Phe-Arg-7-amido-4-methylcoumarin, Hydrochloride-Calbiochem*) and 86 μL of buffer (0.1 M Tris-HCl, 0.15 M NaCl and 0.1 M Triton X-100) at pH 8.0 was performed. After 20 minutes of incubation at 37 °C, readings were taken for 30 minutes (at 5-minute intervals) with a spectrophotometer (*Biotek - Synergy HT*) using the following parameters: Sensitivity 65; “Optic exposition - Top”; Wavelength 380/20 (excitation) and 460/40 (emission).
