# Author Correction: Effects of the serine protease inhibitor rBmTI-A in an experimental mouse model of chronic allergic pulmonary inflammation

**DOI:** 10.1038/s41598-019-53742-w

**Published:** 2019-11-13

**Authors:** Ariana Corrêa Florencio, Robson S. de Almeida, Fernanda M. Arantes-Costa, Beatriz M. Saraiva-Romanholo, Adriana F. Duran, Sérgio D. Sasaki, Mílton A. Martins, Fernanda D. T. Q. S. Lopes, Iolanda F. L. C. Tibério, Edna A. Leick

**Affiliations:** 1 Department of Medicine, School of Medicine, University of São Paulo, São Paulo, SP Brazil; 2 Natural and Human Sciences Center, Federal University of ABC, Santo André, SP Brazil

Correction to: *Scientific Reports* 10.1038/s41598-019-48577-4, published online 02 September 2019

The Acknowledgements section in this Article is incorrect.

“This study was supported by the following Brazilian Scientific Agencies: Coordenação de Aperfeiçoamento de Pessoal de Nível Superior (CAPES), Fundação de Amparo à Pesquisa do Estado de São Paulo (FAPESP), Conselho Nacional de Desenvolvimento Científico e Tecnológico (CNPq), and Faculdade de Medicina da Universidade de São Paulo - Brazil (LIM-20-HC-FMUSP). The authors declare no competing financial interests.”

should read:

“This study was supported by the following Brazilian Scientifc Agencies: Coordenação de Aperfeiçoamento de Pessoal de Nível Superior (CAPES), Conselho Nacional de Desenvolvimento Científico e Tecnológico (CNPq), and Faculdade de Medicina da Universidade de São Paulo - Brazil (LIM-20-HC-FMUSP). This study also received the support from São Paulo Research Foundation (FAPESP), grant #2009/53904-9. The authors declare no competing financial interests.”

